# Successful surgical intervention for delayed chylopericardial tamponade following aortic valve replacement: a case report

**DOI:** 10.1186/s13019-014-0190-9

**Published:** 2014-11-30

**Authors:** Takayuki Gyoten, Toshio Doi, Kazuaki Fukahara, Naoki Yoshimura

**Affiliations:** Department of Cardiovascular Surgery, JA Nagano Koseiren Shinonoi General Hospital, Japan 666-1 Shinonoiai, Nagano, 388-8004 Nagano, Japan; First Department of Surgery, University of Toyama, 2630 Sugitani, Toyama, 930-0194 Japan

**Keywords:** Chylopericardium, Cardiac tamponade, Thoracic duct

## Abstract

Chylopericardium is one of the rarest complications of cardiac surgery. It is related to direct or indirect injury to the thoracic duct or its tributaries and can result in serious morbidity or even death. A 77-year-old Japanese woman who had severe aortic stenosis underwent routine aortic valve replacement. At 15 days after surgery, she developed cardiac tamponade, which was caused by chylopericardium. She was successfully treated by direct clipping of a thoracic duct tributary and mass ligation of the thymus. This rare condition needs to be kept in mind as a possible cause of postoperative cardiac tamponade.

**Electronic supplementary material:**

The online version of this article (doi:10.1186/s13019-014-0190-9) contains supplementary material, which is available to authorized users.

## Background

Chylopericardium is a rare complication of cardiac surgery, being reported in 0.25 to 0.5% of patients [1]. Intrathoracic leakage of lymph after cardiac surgery usually results from disruption of the thoracic duct or one of its major tributaries [2]. Uncontrolled leakage of lymph can cause hypoproteinaemia, malnutrition, immune deficiency, and cardiac complications such as pericarditis or cardiac tamponade [2].

We report a patient with delayed chylopericardial tamponade who was treated by ligation of a thoracic duct tributary and thymic mass ligation.

## Case presentation

A 77-year-old Japanese woman who had severe aortic stenosis underwent routine aortic valve replacement (AVR) with a 19-mm bioprosthetic valve (Trifecta Valve™; St. Jude Medical, St. Paul, MN) via median sternotomy. The thymus was not excised, but the isthmus was split in the midline by electrocautery. Minimal aortic dissection was performed for cross-clamping, but pulmonary artery dissection was not done. There were no intraoperative complications and the mediastinal drains were removed on the following morning after drainage of 80 mL of serous fluid.

The patient was scheduled to be discharged 15 days after surgery because she had chosen to undergo cardiac rehabilitation. On the 15^th^ postoperative day, she developed progressive shortness of breath that was accompanied by nausea and vomiting. She was hypotensive, with a systolic pressure of 65 mmHg, and the jugular venous pressure was elevated. Echocardiography demonstrated a 2 cm pericardial effusion with diastolic right ventricular collapse, so cardiac tamponade was diagnosed. A pericardial pigtail catheter was inserted, and 500 mL of milky white fluid was drained with immediate hemodynamic improvement. This fluid was lymph with a high triglyceride concentration (2.95 × 10^4^ mg/L), so chylopericardium was diagnosed. Drainage from the pericardial catheter continued for 2 weeks (60 to 800 ml daily), while she was managed conservatively with a high protein, low fat diet containing medium-chain triglycerides. Then the patient was fasted for 2 weeks with total parenteral nutrition and subcutaneous somatostatin (100 μg three times daily).

Her serum total protein and albumin levels and body weight decreased substantially during this period, while drainage of lymph persisted and cardiac tamponade-like symptoms developed immediately after clamping of the drain was attempted. Because conservative management had failed to control the leakage of lymph, surgery was performed on the 43rd postoperative day (see Additional file 1).

**Figure 1:**
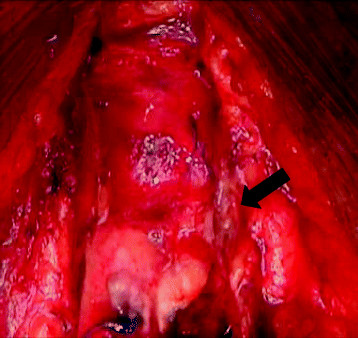
**Accumulation of lymph on the surface of the thymus (arrow).**

**Figure 2:**
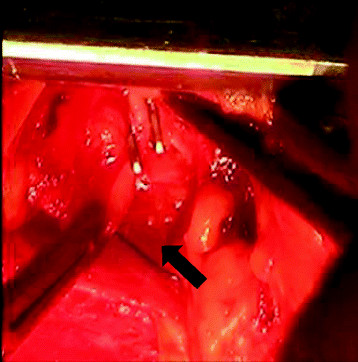
**Clipping of the lymphatic running above the innominate vein (arrow).**

One hundred milliliters of thick cream (50 ml of concentrated milk (4.6% fat) and 50 ml of fresh cream (47% fat)) was introduced into the stomach 2 hours before starting the operation to facilitate identification of the thoracic duct and lymphatics. After sternotomy was repeated via the same incision, two leaks were identified. One leak was on the transected surface of the left lobe of the thymus and this was oversewn with 3-0 Prolene (Figure 1). The other leak was from a lymphatic running directly above the innominate vein and this was clipped (Figure 2). After these procedures, no further leakage of lymph was seen in the surgical field. A pericardial window was created before finishing the operation so that lymph would not accumulate in the pericardium if another leak occurred.

Postoperative chest radiography and echocardiography confirmed no re-accumulation of pericardial fluid and the patient was discharged 4 weeks after the second operation.

## Discussion

It is unusual for the thoracic duct to be injured during AVR via median sternotomy because the duct runs outside the operative field. Occurrence of chylous pericardial effusion more than 2 weeks after aortic valve replacement seems to be highly unusual.

After cardiac surgery, chylopericardium is generally caused by disruption of thoracic duct tributaries rather than the main duct itself [1]. These tributaries have a variable intra-thoracic course and are found in the pericardial reflections and thymic tissue. Leakage of lymph due to disruption of a lymphatic running above the innominate vein has not been reported before, although leakage from the transected thymus has been documented [1].

Initial treatment of chylopericardium is generally conservative, consisting of pericardiocentesis, dietary manipulation, and infusion of somatostatin [3]. There is no consensus about the indications for surgery in patients with chylopericardium. Although some authors have achieved success with conservative treatment of chylopericardium after cardiac surgery [2],[4], Chadi et al. reported that conservative management failed in 57.1% of patients, while surgery was always curative [5]. Early surgery is needed for hemodynamically significant uncontrolled chylopericardium, which has a high mortality and morbidity related to nutritional, metabolic, and immunologic abnormalities, as well as cardiac complications.

Standard surgery is performed via open thoracotomy or video-assisted thoracoscopy, and involves thoracic duct mass ligation above the diaphragm with creation of a pericardial window [5]. In our patient, direct clipping of a lymphatic running above the innominate vein and thymic ligation stopped the leakage of lymph, so we completed surgery without ligating the thoracic duct. There is a risk of insufficient lymph drainage and lower limb edema after thoracic duct ligation, so it is preferable to avoid it if possible. Leakage of lymph does not always occur from a single site and may even develop at a site unrelated to the previous surgical field. In the present case, leakage was detected at 2 sites and chylopericardium was eliminated by treating these leaks. If the site of lymphatic leakage cannot be identified when the surgical field is reopened, ligation of the thoracic duct is the second choice. However, abundant leakage may be readily identifiable in patients like ours with chylopericardium causing cardiac tamponade.

## Conclusions

The most likely mechanism of injury to the lymphatic running above the innominate vein in our patient was traction during manipulation of the heart and aorta, while leakage after thymic transection might have occurred because electrocautery was not effective since thin-walled lymphatics contain little coagulable material [1].

In conclusion, surgical intervention may be required for medically uncontrollable chylopericardium after cardiotomy. Careful retraction and treatment of the transected thymus are important to prevent leakage of lymph and chylopericardium should be remembered as a possible cause of postoperative cardiac tamponade.

## Consent

Written informed consent was obtained from the patient for publication of this case report and any accompanying images. A copy of the written consent is available for review by the Editor-in-Chief of this journal.

## Additional file

## Electronic supplementary material

Additional file 1: Timeline. (DOCX 15 KB)

Below are the links to the authors’ original submitted files for images.

Authors’ original file for figure 1

Authors’ original file for figure 2
