# Supplementary material for: Successful surgical intervention for delayed chylopericardial tamponade following aortic valve replacement: a case report
Source: J Cardiothorac Surg. 2014 Nov 30;9:190. doi: 10.1186/s13019-014-0190-9 (PMC4255646; doi:10.1186/s13019-014-0190-9)
Supplement: Supplementary file 1 — Additional file 1: Timeline. (DOCX 15 KB) [file 13019_2014_190_MOESM1_ESM.docx]

Timeline

2002: Commenced medical treatment for diabetes mellitus, hyperlipidemia, and hypertension.

2012: Aortic valve stenosis was diagnosed.

2013 (11/8): Aortic valve replacement was performed.

2013 (11/23): Chylopericardial tamponade was diagnosed.

2013 (12/21): Surgical intervention for chylopericardial tamponade (direct clipping of a lymphatic and thymic ligation).
