# Supplementary figures and images for: Successful surgical intervention for delayed chylopericardial tamponade following aortic valve replacement: a case report
Source: J Cardiothorac Surg. 2014 Nov 30;9:190. doi: 10.1186/s13019-014-0190-9 (PMC4255646; doi:10.1186/s13019-014-0190-9)

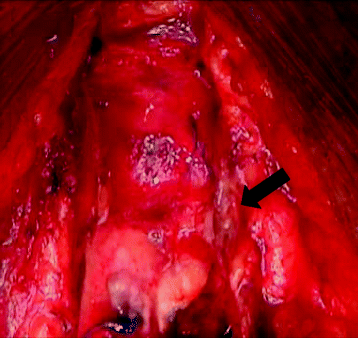

Supplement: Supplementary file 2 — Authors’ original file for figure 1 [file 13019_2014_190_MOESM2_ESM.gif]

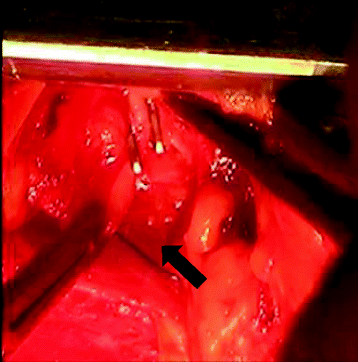

Supplement: Supplementary file 3 — Authors’ original file for figure 2 [file 13019_2014_190_MOESM3_ESM.gif]
